# Distance-quality trade-off and choice of family planning provider in urban Pakistan

**DOI:** 10.1093/inthealth/ihac063

**Published:** 2022-09-28

**Authors:** Imtiaz Hussain, Sidrah Nausheen, Arjumand Rizvi, Uzair Ansari, Mir Baz, Kaneez Zehra, Sahar Yameen, Kristy Hackett, Zohra Lassi, David Canning, Iqbal Shah, Sajid Bashir Soofi

**Affiliations:** Centre of Excellence for Women and Child Health, Aga Khan University, Karachi 74800, Pakistan; Department of Obstetrics and Gynecology, Aga Khan University, Karachi 74800, Pakistan; Centre of Excellence for Women and Child Health, Aga Khan University, Karachi 74800, Pakistan; Centre of Excellence for Women and Child Health, Aga Khan University, Karachi 74800, Pakistan; Centre of Excellence for Women and Child Health, Aga Khan University, Karachi 74800, Pakistan; Centre of Excellence for Women and Child Health, Aga Khan University, Karachi 74800, Pakistan; Centre of Excellence for Women and Child Health, Aga Khan University, Karachi 74800, Pakistan; Harvard T. H. Chan School of Public Health, Harvard University, Boston, MA 02115, USA; Centre of Excellence for Women and Child Health, Aga Khan University, Karachi 74800, Pakistan; Harvard T. H. Chan School of Public Health, Harvard University, Boston, MA 02115, USA; Harvard T. H. Chan School of Public Health, Harvard University, Boston, MA 02115, USA; Centre of Excellence for Women and Child Health, Aga Khan University, Karachi 74800, Pakistan; Department of Paediatrics and Child Health, Aga Khan University, Karachi 74800, Pakistan

**Keywords:** cross-sectional survey, distance of facility, family planning, quality of services, urban Pakistan

## Abstract

**Background:**

There is limited evidence between contraceptive use, availability of commodities and distance to the facility in developing countries. Distance to the facility is an essential determinant of contraceptive use. Still, women may not seek family planning services from the nearest facility and may be prepared to travel the farthest distance to receive quality family planning services.

**Methods:**

We analyzed women's survey data linked to health facility data and applied an alternate specific conditional logit model to examine the distance a woman is prepared to travel and the quality of services offered by facilities in urban areas in Karachi, Pakistan.

**Results:**

This study analyzed data from 336 women and 28 facilities and identified that the mean distance to the nearest facility was 0.44 km; the chosen facility was, on average, 5 km away. Women preferred facilities that offered a range of contraceptive methods and additional services provided by female healthcare providers only. Furthermore, on average, women are willing to travel a further 1.7 km for a facility that offers more family planning methods, 1.4 km for a facility that offers additional health services and 11 km for a facility that offers services delivered by female healthcare providers.

**Conclusions:**

The findings highlight the quality measures women prioritize over distance and consider essential when choosing a family planning facility.

## Introduction

Globally, among 1.9 billion women of reproductive age, 1.1 billion have family planning needs; 840 million are using contraceptive methods, but 270 million have an unmet need for contraception worldwide.^1^ Family planning is essential for reducing maternal mortality in low- and middle-income countries (LMICs), which contributes 99% of maternal deaths.^2^ The importance of family planning was further reiterated in the 1994 International Conference on Population and Development for women's health. A 2012 analysis of contraceptive use in 172 countries identified a 44% reduction in maternal deaths and a 29% reduction in maternal deaths by satisfying the unmet need for contraception alone.^2^ In LMICs where the number of unwanted pregnancies, unmet need for contraceptives and maternal deaths are still high, increasing the number of contraceptive users could help to resolve this issue.

Pakistan is the sixth most populous country globally, with a high total fertility rate (i.e. 3.6 births per woman).^3^ According to the Pakistan Demographic and Health Survey 2017–2018, modern contraceptive use among married women aged 15–49 y was 25%, while 17% of married women have an unmet need for family planning. Moreover, discontinuation rates are high, especially for the intrauterine device. A study reported that more than half (56.5%) of those women do not switch to other methods.^4^ The country launched its first family planning program in 1960 before introducing the Lady Health Worker Program. However, even 60 y after its inauguration, the government still struggles to increase modern contraceptive uptake. To overcome this challenging situation, Pakistan committed to family planning's 2020 goal of achieving universal access to reproductive health by increasing the contraceptive prevalence rate to 55% by 2020.^5^

Earlier studies have identified social, cultural, economic and environmental factors such as age, education, religion, traditional values and beliefs, socioeconomic status, women's status and autonomy, location and level of knowledge that influence family planning methods.^6–11^ Among all those factors, distance from the facility has been shown to control a wide range of healthcare utilization.^12,13^ Facility choice for healthcare utilization is complex and depends on geographical factors, household wealth and hours to travel. Studies have shown that distance to the facility is directly associated with the quality of healthcare services. Women may not necessarily seek health services from the nearest facility; instead, they might seek care from facilities of better quality, even if these are located farther away.^14,15^ For example, a recent study from Tanzania reported that the average distance traveled to a facility for family planning services was 2.9 km away, even although the nearest facility was only 1.2 km away, and only 33% of the women accessed the nearest facility.^15^ A study from Vietnam reported that quality of care at the initiation of family planning determines the continuation of family planning at the follow-up. Almost two-thirds of the users continued using family planning when they received medium- to high-quality family planning services.^16^

Similarly, providers' knowledge and competency, availability of contraceptive methods, maintaining privacy and confidentiality and shorter waiting times are positively associated with family planning methods.^17^ This evidence shows that distance is not a barrier when the quality of care matters. Therefore, our study also intends to determine the distance a woman is willing to travel to use a healthcare facility with better quality family planning services in urban settings in Karachi, Pakistan. Understanding this would help formulate policies to increase contraception uptake by improving the quality of services provided by the health facilities in Pakistan.

## Methods

The study draws on data from a baseline cross-sectional survey of the Willows Program. This community-based reproductive health trial provides family planning information, education and referral to married women of reproductive age (MWRA) through household visits in Karachi's underserved urban areas, Pakistan. The detailed methodology has been described elsewhere.^18^

This study was conducted from December 2017 to June 2018 in Jamshed town (intervention area) and Yousaf Goth (control area) in Karachi, Pakistan. The study used two primary data sources, that is, a reproductive health survey among a representative sample of MWRA and a facility health survey that provides family planning services and products in selected areas. The reproductive health survey involved MWRA aged 16–44 y who were residents of those urban towns, usual household members, spoke at least one of the four commonly spoken languages (Urdu, Pushto, English or Sindhi) and self-reported themselves as fertile. In the reproductive health survey, MWRA were particularly asked if they had ever used or were currently using any family planning method. Those who said yes were further asked the name and type of facility where they received their last services. MWRA who had ever used or were currently using any method of contraception from a health facility in a surrounding area were included in the analysis. Additionally, facilities that were most commonly reported (i.e. by ≥5 women) were surveyed.

A sample size of 1836 (∼2000) from each intervention and control area was required for a parent study, assuming an estimated modern contraceptive prevalence rate of <30% in selected regions. A three-stage random sampling design was carried out in STATA using a uniform [0,1] random number generator with a fixed seed for a reproductive health survey. First, 110 clusters from each site were randomly selected. Second, the household listing was developed to choose households with MWRA randomly. Finally, if >1 MWRA lived in a chosen family, we randomly selected one from home in the third stage. From 4210 MWRA identified, 1612 women reported ever or current use of a family planning method from facilities. We identified the 28 most commonly reported health facilities from 336 MWRA that were finally included in the analysis. The 28 health facilities surveyed included hospitals, family planning or health centers and dispensaries.

Data were collected through face-to-face interviews with eligible women using a structured tablet-based questionnaire on the CommCare application. MWRAs were asked a range of reproductive health questions, including sociodemographic characteristics. The primary variable of interest was the facility choice for the quality indicator, where the value of 1 is utility and 0 means no utility. Route layer calculator—ArcGIS Network Analyst extension was used to calculate the road distance from each woman's house to the facility.^19^ The distance was calculated between the closest facility and the chosen facility. Distances calculated were the quickest and shortest routes; traffic and seasonal characteristics were not defined in the route analysis.

The explanatory variables included a woman's age, education (no education, primary, secondary and above), the type of health facility visited (hospital, family planning /health center, dispensary) and wealth index (poorest, poor, middle, richer and richest). The wealth index was constructed using principal component analysis with various socioeconomic factors, including household construction, assets, utilities, source of drinking water and sanitation facilities. Health facility variables (i.e. quality indicators) included several services, number of family planning methods, fees for family planning services at the facility, days on which those facilities are functional (weekdays only, and weekdays and weekends) and service providers at the facility (only female providers, and both female and male providers).

In the facility survey, a representative from each health facility was asked what contraceptive methods they are providing to their clients from the given list. Interviewers also checked each type of contraception the facilities have in stock and validated the expiry dates on the stock. Family planning methods provided to clients were only considered when they were available in the stock (within their expiry dates) at the facility on the survey day.

Descriptive statistics were presented using frequency and percentage for categorical variables and mean with SD for continuous variables. Because the study treated the choice of facility utilization as unconditional, an alternate specific conditional logit model was used, which has previously been used to predict the selection of the facility in the Willow's Program in Tanzania.^15^ Based on the assumption that a woman would choose the type of facility which will allow her the highest quality, this model allocates a woman a utility. For example, the utility for a woman “i” from choosing facility “j” is given as follows:


\begin{eqnarray*} {u}_{ij} = \sum\limits_{m = 1}^M {{\beta }_m{x}_{ijm}} + \sum\limits_{j = 1}^J {\sum\limits_{p = 1}^P {{\lambda }_{jp}} } {Z}_{ip} + {\varepsilon }_{ij} \end{eqnarray*}

The equation depicts M number of variables, including measures of quality of facility and distance that vary across woman and facility that affect her utility and has been explained in detail elsewhere.^15^

## Results

The sociodemographic characteristics are presented in Table 1.

**Table 1.** Sociodemographic characteristics of women visiting the facility (n=336)

Sociodemographic characteristics	mean±SD
Age (y)	33.21±5.91
Age (y)	n (%)
17–19	1/336 (0.3%)
20–24	27/336 (8.0%)
25–29	60/336 (17.9%)
30–34	95/336 (28.3%)
35–39	88/336 (26.2%)
40–44	65/336 (19.3%)
Education	
No education	119/336 (35.4%)
Primary	53/336 (15.8%)
Secondary	123/336 (36.6%)
Above secondary	41/336 (12.2%)
Wealth index—comparable with DHS	
Poorest	31/333 (9.3%)
Poorer	67/333 (20.1%)
Middle	128/333 (38.4%)
Richer	84/333 (25.2%)
Richest	23/333 (6.9%)
Facility type visited	
Hospital	221/336 (65.8%)
FP center/health center	111/336 (33.0%)
Dispensary	4/336 (1.2%)
Distance to (km)	mean±SD
Hospital	10.09±7.28
FP center/health center	9.81±7.82
Dispensary	9.56±8.02
Distance to the facility chosen (km)	5.08±6.00
Distance to the nearest facility (km)	0.44±0.41

DHS, demographic health survey; FP, family planning.

The mean age of MWRA was 33 y and more than half (54.5%) were in the 30–39 y age bracket; one-third (35.4%) had no education and belonged to the median wealth index (38.4%). Almost two-thirds of MWRA visited a hospital on average 10 km away, while one-third visited a family planning/health center, which was 9.8 km away. The mean distance to the nearest facility was 0.44 km, while the chosen facility was 5 km away.

**Figure 1.**
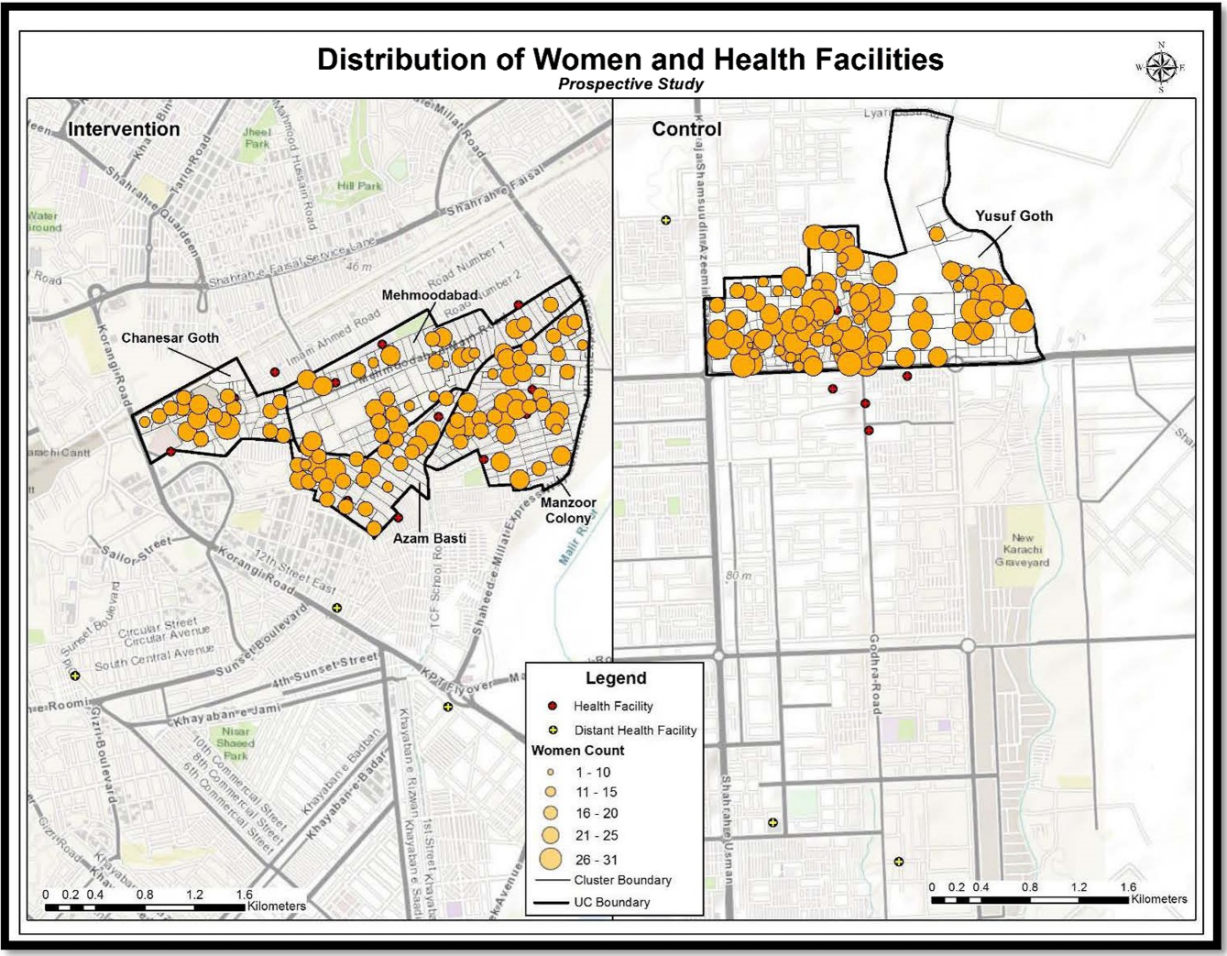
Distribution of women and health facilities in the study area.

Figure 1 shows the spatial distribution of women and health facilities in the study area (intervention and control separately). Of the 28 health facilities surveyed, 12 were hospitals, 15 were family planning/health centers and one was a dispensary.

**Figure 2.**
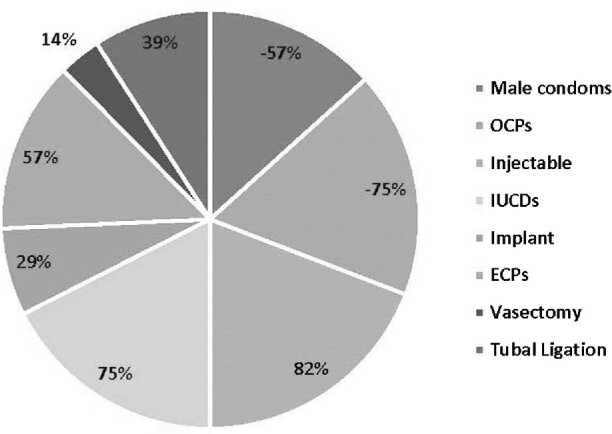
Percentages of family planning methods provided at health facilities of urban Karachi (n=28).

The distribution of quality indicators by health facility are presented in Table 2 and family planning methods available in each facility are illustrated in Figure 2. The mean numbers of family planning methods and other services provided in those facilities were four and five, respectively. More than half (57.14%) of all facilities charged a fee for their services, were open 7 d a week (61%) and had both female and male providers (57%).

**Table 2.** Distribution of quality indicators by facility type

	Hospital^a^	FP center/health center^b^	Dispensary	Total
	N=12	N=15	N=1	N=28
Number of FP methods provided	4.33±2.57	4.20±1.86	5.00±0.0	4.29±2.12
Number of services other than FP provided	5.92±3.12	4.13±2.07	2.00±0.0	4.82±2.70
Fees for family planning services at facility	8 (66.67%)	7 (46.67%)	1 (100%)	16 (57.14%)
Facility open days				
Weekdays only	4 (33%)	7 (47%)	0 (0%)	11 (39%)
Both weekdays and weekends	8 (67%)	8 (53%)	1 (100%)	17 (61%)
Providers assigned to facility				
Only female providers available	5 (42%)	7 (47%)	0 (0%)	12 (43%)
Both male and female providers available	7 (58%)	8 (53%)	1 (100%)	16 (57%)

Data are presented as mean±SD for continuous measures and as n/total (%) for categorical measures.

a including government and private hospitals.

b including private clinic, family planning (FP) center or government and private hospitals.

We applied a conditional logit model of facility choice in Table 3. The coefficients can be interpreted as the effect of each facility's characteristics on women's utility. The distance to the facility is reported in km, and a dummy for being the facility closest to the women as a possible factor influencing utility and choice. The negative coefficient for the distance indicates that the farther away a health facility was from a woman's home, the less likely it was to have been selected. Being closer to the facility, the more likely it is to be chosen by a woman. Health facilities that provided more family planning methods were more likely to be selected by a woman; similarly, health facilities that offered other healthcare services were more likely to be chosen by a woman. Moreover, health facilities that provided services delivered by female providers were more likely to be selected by a woman. No difference was observed for the choice of a facility in terms of its type, service fees or working days.

**Table 3.** Determinants for the choice of utility using multivariable analysis

	Multivariate (shortest distance)				
Indicator	Coef.		95% CI		p
Distance to health facility (km)	−0.17	−0.20		−0.13	<0.0001
Nearest facility	1.16	0.59		1.73	<0.0001
Number of FP methods provided	0.27	0.03		0.52	0.030
Number of services other than FP provided	0.23	0.07		0.39	0.006
Facility type					
Hospital	0.90	−0.25		2.06	0.124
Other (FP center/dispensary)	Ref				
Fees for family planning services at the facility (ref=no)	0.21	−1.11		1.53	0.757
Facility open days					
Weekdays only	Ref				
Weekends and weekdays	0.43	−0.65		1.52	0.434
Providers assigned to the facility					
Male and female providers	Ref				
Only female providers	1.89	0.59		3.20	0.004

Women's education and wealth index were used as individual level covariates in the conditional logit model. FP, family planning.

Results in Table 4 show the estimated extra distance a woman is willing to travel to a health facility with better quality indicators. For example, the results show that a woman is willing to travel 1.7 km for a facility that offers more family planning services, 1.4 km for a health facility that provides additional services and 11.4 km for one that offers services delivered by female providers only.

**Table 4.** Shortest distance traveled by women for an increased quality indicator

Estimated extra distances (in km) women are willing to travel for an increased quality indicator at the average values of other covariates	
Indicator	Shortest distance
Number of FP methods provided	1.7
Number of services other than family planning provided	1.4
Providers assigned to a facility with only female providers	11.4

FP, family planning.

## Discussion

This study used health facility data linked to women's survey data to examine the distance a woman is prepared to travel and the association of indicators of health facility quality and women's choice of family planning facility. The existing literature indicates that proximity to healthcare facilities influences healthcare utilization. Still, our study indicates that women prefer to attend those facilities that offer more family planning methods as well as healthcare services other than family planning. They also picked a facility where service providers are female without considering distance as a barrier. A recent study from Tanzania has reported that women were willing to travel extra miles to receive services from facilities that offer more family planning methods, do not charge a fee, have fewer stock-outs and provide fewer health services.^15^ Our results also imply that women are willing to bypass the nearest facilities and are prepared to travel up to 11 km to receive services from health facilities they prefer, in particular a health facility that offers quality services and a wide range of methods. A study from Ethiopia^20^ also reported that the number and range of methods available (method choice) are independently associated with contraceptive utilization in rural Ethiopia. Similarly, the quality measure of the customer-client relationship was found to be a positive predictor of contraceptive use in Kenya.^21^ Another study from urban Kenya reported that women are willing to travel extra distances to secure greater choice regarding family planning methods and complementary services.^22^ Our findings also suggest that the choice of facility depends on the availability of female healthcare providers. This preference is mainly for the insertion of the intrauterine contraceptive device than for other methods. In Pakistani society, many women prefer receiving reproductive healthcare services from female care providers for sociocultural reasons. A study from Pakistan also reports that the traditional norms of modesty of Muslim women limits their access to healthcare facilities for contraception and that they seek a female healthcare provider.^23^ This cultural aspect needs to be explored in detail as limited evidence is available in the literature.

Although a recent study from Tanzania^15^ reported that women prefer to receive family planning services at health centers rather than hospitals, but prefer hospitals over dispensaries because of the availability of more family planning methods, less fees and less stock-out, this finding was not evident in our study. In our study, women were willing to receive services from any health facility provided they offer quality healthcare services. Our study also showed that family planning service fees were not a barrier to prioritizing health facilities, indicating that women considered “quality” a priority and were ready to accommodate all associated expenses to receive quality services.

To the best of our knowledge, this is the first study from the South Asian region to assess the distance a woman is willing to travel to receive a good quality family planning service. The results will prove instrumental in deciding how to increase the uptake of these services in Pakistan and LMICs.

This study has some limitations. First, all the distances were calculated from the woman's home to the facility in a straight line, which does not account for road conditions and transportation issues, especially in an urban area like Karachi. It is also possible that the facility the woman used was not near her home, but was instead near her workplace, school or another place she frequently visited. Therefore, this study did not account for those distances and might not accurately reflect the actual distance and choice. Second, women not currently using a contraceptive method were not asked when they last used a contraceptive method. It is possible that some of the health facilities were newly built and did not exist at that time. Third, the study only measured the quality of the facilities that most women preferred. However, we did not observe pharmacies/drug stores where family planning methods were available because they are mostly run without the supervision of clinicians/healthcare providers and carry no additional services. Fourth, this study measured some specific quality indicators. Other quality measures, such as follow-up for family planning services, stock-out of family planning commodities, client-provider interactions and sufficient time for counseling and privacy, received an insufficient number of responses. Fifth, the road conditions, structures, facilities, availability of services and sociodemographic factors differ in rural and urban areas, as rural areas have their own dynamics. However, the findings of this study can be generalized to other urban settings of Pakistan and LMICs.

## Conclusion

The current study highlights that women are prepared to travel extra distances to receive quality family planning services from facilities that offer more family planning methods, services other than family planning and services delivered by female healthcare providers only. In addition, the findings highlight the quality measures that women prioritize over distance and consider important when they choose a facility for family planning.

The implications for future researchers would be to highlight the quality indicators for contraceptive use, which should be regarded while putting policies into place for family planning. In addition, policymakers should focus on improving the quality of existing healthcare centers and the provision of female healthcare providers, rather than on increasing their number.

## Data Availability

All the data are available from the corresponding author on reasonable request.
